# Manganese and Vanadium Co-Exposure Induces Severe Neurotoxicity in the Olfactory System: Relevance to Metal-Induced Parkinsonism

**DOI:** 10.3390/ijms25105285

**Published:** 2024-05-13

**Authors:** Hilary Afeseh Ngwa, Alejandra Bargues-Carot, Huajun Jin, Vellareddy Anantharam, Arthi Kanthasamy, Anumantha G. Kanthasamy

**Affiliations:** 1 Iowa Center for Advanced Neurotoxicity, Department of Biomedical Sciences, Iowa State University, Ames, IA 50010, USA; 2 Isakson Center for Neurological Disease Research, Department of Physiology and Pharmacology, University of Georgia, Athens, GA 30602, USA; abargues@uga.edu (A.B.-C.); huajun.jin@uga.edu (H.J.); vellareddy.anantharam@uga.edu (V.A.);

**Keywords:** alpha-synuclein, metal mixtures, vanadium, manganese, neurotoxicity, Parkinsonism

## Abstract

Chronic environmental exposure to toxic heavy metals, which often occurs as a mixture through occupational and industrial sources, has been implicated in various neurological disorders, including Parkinsonism. Vanadium pentoxide (V_2_O_5_) typically presents along with manganese (Mn), especially in welding rods and high-capacity batteries, including electric vehicle batteries; however, the neurotoxic effects of vanadium (V) and Mn co-exposure are largely unknown. In this study, we investigated the neurotoxic impact of MnCl_2_, V_2_O_5,_ and MnCl_2_-V_2_O_5_ co-exposure in an animal model. C57BL/6 mice were intranasally administered either de-ionized water (vehicle), MnCl_2_ (252 µg) alone, V_2_O_5_ (182 µg) alone, or a mixture of MnCl_2_ (252 µg) and V_2_O_5_ (182 µg) three times a week for up to one month. Following exposure, we performed behavioral, neurochemical, and histological studies. Our results revealed dramatic decreases in olfactory bulb (OB) weight and levels of tyrosine hydroxylase, dopamine, and 3,4-dihydroxyphenylacetic acid in the treatment groups compared to the control group, with the Mn/V co-treatment group producing the most significant changes. Interestingly, increased levels of α-synuclein expression were observed in the substantia nigra (SN) of treated animals. Additionally, treatment groups exhibited locomotor deficits and olfactory dysfunction, with the co-treatment group producing the most severe deficits. The treatment groups exhibited increased levels of the oxidative stress marker 4-hydroxynonenal in the striatum and SN, as well as the upregulation of the pro-apoptotic protein PKCδ and accumulation of glomerular astroglia in the OB. The co-exposure of animals to Mn/V resulted in higher levels of these metals compared to other treatment groups. Taken together, our results suggest that co-exposure to Mn/V can adversely affect the olfactory and nigral systems. These results highlight the possible role of environmental metal mixtures in the etiology of Parkinsonism.

## 1. Introduction

Among potentially toxic elements, heavy metals, including copper (Cu), lead (Pb), manganese (Mn), vanadium (V), and cadmium (Cd), are ubiquitous in nature, and some are essential for a wide range of biological processes, such as maintaining cell structure and regulating metabolic function, gene expression, neurotransmission, and antioxidant responses [1]. However, the excessive accumulation of metals in the nervous system can have harmful effects, resulting in deficits in neurodevelopment, neurobehavior, and cognition, and may cause neurodegeneration depending on the valence states, metal exposure route and duration, bioavailability, chemical forms, and the subject’s life stage [2]. Various metals, including Cd, chromium, nickel, Pb, and Mn, have been shown to alter the ability to smell following chronic exposure [3]. Other studies have reported olfactory dysfunction in cohorts of professional welders [4,5]. The epidemiological, experimental, and clinical evidence indicates a strong correlation between exposure to toxic metals and various neurological diseases, including Alzheimer’s disease, Parkinson’s disease (PD), and amyotrophic lateral sclerosis [2,6]. Currently, no clinically efficacious treatments exist for individuals suffering from chronic neurotoxicity induced by recurrent exposure to toxic metals. Although the exact molecular events remain unclear, the cellular mechanisms underlying toxic heavy metal-induced neurotoxicity likely involve oxidative stress, lipid peroxidation, mitochondrial dysfunction, protein misfolding, apoptosis, DNA damage, the disruption of ATP synthesis, and epigenetic and genetic alterations [7,8].

Among the toxic heavy metals associated with neurological diseases, Mn receives much interest, given its ability to induce manganism, which is characterized by a severe neurological deficit that often resembles the involuntary extrapyramidal symptoms associated with PD [9,10,11,12,13]. Chronic Mn neurotoxicity occurs often from inhalation in occupational settings, including welding, mining and mineral processing, metal manufacturing emissions, fossil fuel combustion, and dry-cell manufacturing [11,14,15,16,17]. The current inhalation reference concentration (RfC) for Mn is 0.05 µg/m^3^ (USEPA, 1993). Mn toxicity is most commonly associated with occupational exposure to aerosols or dust containing extremely high levels of Mn (>1–5 mg/m^3^) [18,19,20,21,22,23,24,25]. Mn levels in aerosols, dust, and fumes can be 200–6000 times higher than the RfC. The basal ganglia, which includes the striatum (STR), is a critical target during Mn exposure [26,27,28,29,30]. One of the features distinguishing manganism from PD is the globus pallidus being more severely affected than all the other regions of the brain during the disease pathogenesis of manganism [31]. Mn-induced striatal dopamine (DA) depletion, which is one of the pathological hallmarks of PD, has also been demonstrated in both in vivo [32] and in vitro [33] studies. Dorman et al. [34] showed the accumulation of MnSO_4_ in the STR and olfactory bulb (OB) of rats following inhalation exposure. V is typically found in the environment as vanadium pentoxide (V_2_O_5_) and is often associated with Mn, particularly in welding rods. It is a significant trace element in fossil fuels, the burning of which results in considerable environmental exposure to V. V compounds are widely utilized in numerous industrial applications, including car and aircraft manufacturing, temperature-resistant alloy production, sulfuric acid, and phthalic anhydride manufacturing, pesticide production, and pigment and paint manufacturing, among others [5,8,9]. The increasing use of V in high-capacity battery manufacturing for energy storage and Li-ion batteries in electric cars has also accelerated its widespread demand. The processing of V slag (about 120 g V_2_O_5_ per kg) is characterized by the formation of dust, with V concentrations ranging from 5 to 120 mg/m^3^ [35]. Crude oil from Venezuela is believed to have the highest V concentrations of 1400 mg/kg. Elevated levels of airborne V (4.7 mg/m^3^) have been found in the breathing zone of steel industry workers [36]. V has been reported in the blood, feces, and urine of workers exposed to airborne V_2_O_5_ dust (USEPA, 1987). ICP-MS analyses of roadside dust samples revealed aluminum (55,090 μg/g), V (70 μg/g), Mn (511 μg/g), and iron (Fe) (21,600 μg/g). The ratio of V to Mn in inhaled dust during occupational exposures can vary from 1:1 to 1:8. Despite V’s extensive industrial use, little is known regarding its adverse neurological effects. Previously, our laboratory reported that low-dose V exposure induces neurotoxic effects in dopaminergic neurons in both cell culture and animal models [37]. Notably, intranasal exposure to low-dose V induces olfactory deficits and damages dopaminergic neurons in the glomerular layer of the OBs [38]. Animal studies have also demonstrated that inhaled V_2_O_5_ damages the nigrostriatal system and other parts of the central nervous system (CNS) [39,40,41]. Inhalation is the primary route of environmental exposure to V, making it a more suitable experimental model for mimicking its exposure [42]. Given that occupationally related V exposure commonly occurs with co-exposure to Mn [43,44,45,46], Mn/V interactive neurotoxicity has become a true concern for industrial workers exposed to metal-containing substances as well as residents and non-workers in the surrounding environments.

We have demonstrated that like many other neurotoxicants associated with Parkinsonism pathogenesis, V and Mn are neurotoxic to dopaminergic neurons through oxidative stress and the PKCδ pro-apoptotic kinase-mediated pathway in in vitro and ex vivo systems [37,47]. Despite what is known about each metal’s neurotoxicity, the neurotoxic response to an Mn/V mixture has not been investigated. In the current study, we investigated the neurotoxic properties of an Mn/V mixture along with that of each metal alone when exposure occurs intranasally in a mouse model. We determined the neurotoxicological responses in the olfactory and nigrostriatal dopaminergic systems following exposure to different treatment groups.

## 2. Results

### 2.1. AP Chronic Intranasal Exposure to Mn, V_2_O_5_, or Their Mixture (Mn/V_2_O_5_) Induces Motor Deficits in Mice

**Figure 1:**
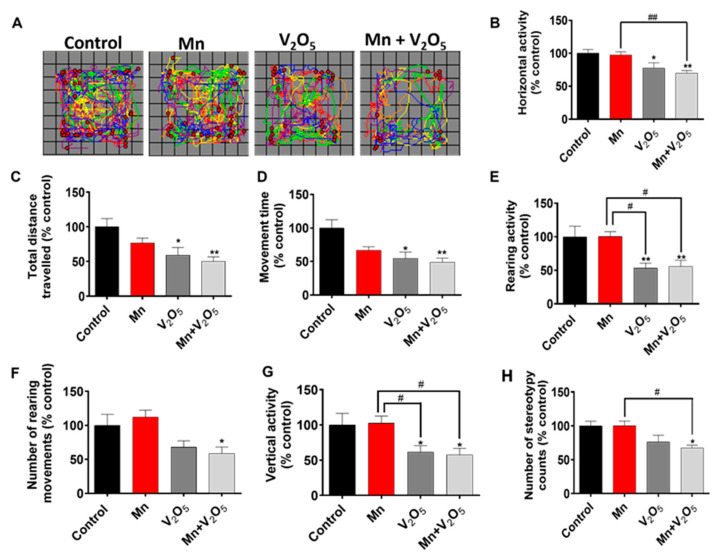
The effects of intranasally administered Mn, V_2_O_5_, and Mn-V_2_O_5_ on locomotor activity. (**A**) Moving track of mice. (**B**), Horizontal activity. (**C**), Total distance traveled. (**D**), Movement time. (**E**), Rearing activity. (**F**), Number of rearing movements. (**G**), Vertical activity. (**H**), Number of stereotypy counts. The vehicle-treated group served as the control, and it was set at 100%. The data represent the mean ± S.E.M. for at least nine animals per group. Asterisks (*, *p* ≤ 0.05; **, *p* < 0.01) indicate significant differences compared with the control unless otherwise indicated, while hashtags (#, *p* ≤ 0.05; ##, *p* < 0.01) indicate significant differences between the indicated treatment groups.

Male C57BL/6 mice were intranasally administered appropriate doses of the metals (Mn and V), both alone and mixed, three times a week for up to one month, as described in Section 4.3. At the end of the one-month treatment period, we evaluated the treatment effects on locomotor activity using a VersaMax automated activity monitor. Figure 1A shows a representative locomotor activity map of the control and treatment groups. Quantitative analysis revealed that the treated mice exhibited significant decreases in various motor parameters compared to the control group (Figure 1B–H). Specifically, V_2_O_5_- and Mn/V_2_O_5_-treated mice exhibited significant decreases in total horizontal movement (Figure 1B); total distance traveled (Figure 1C); total movement time (Figure 1D); rearing activity (Figure 1E); the number of rearing movements (Figure 1F); vertical activity (Figure 1G); and the number of stereotypy counts (Figure 1H) relative to the control group. Notably, the Mn-V co-exposure group showed the most severe locomotor deficits compared to Mn- and V-alone groups. The severity of motor deficits is given in the following order: Mn/V_2_O_5_ > V_2_O_5_ > Mn. Collectively, these results suggest that chronic intranasal exposure to mixed Mn/V induces severe motor dysfunction in mice.

### 2.2. Chronic Intranasal Exposure to Mn, V_2_O_5_, or Their Mixture (Mn/V_2_O_5_) Induces Olfactory Dysfunction in Male Mice

**Figure 2:**
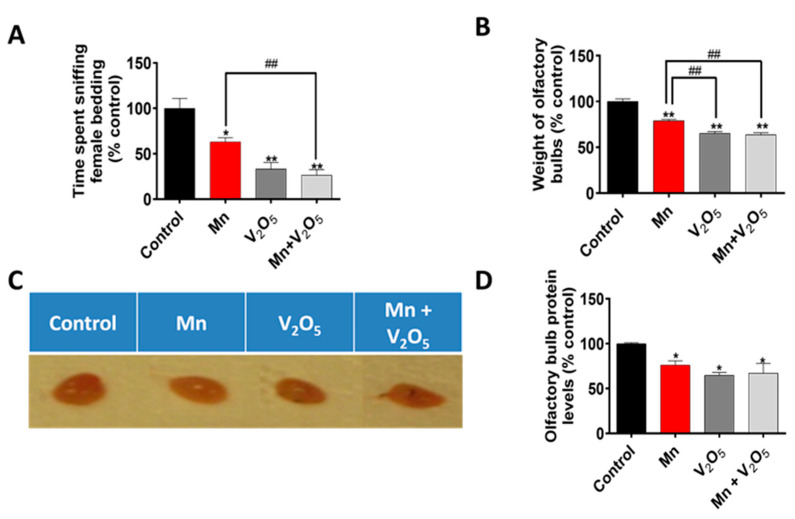
Effects of intranasally administered Mn, V_2_O_5_, and Mn/V_2_O_5_ on pheromonal olfaction (sniffing ability) and the size of mouse OBs. (**A**), Animals were exposed to bedding from female cages at the end of the study, and the amount of time out of three minutes that the animals spent sniffing the bedding was recorded. (**B**), Animals were sacrificed one week following the treatment, and the OBs were dissected in the same manner every time and weighed. (**C**), Snapshot showing the different sizes of the OBs following treatments. (**D**), Graphical representation of the total protein of the homogenized OBs following treatment, as measured with the Bradford assay. The data represent the mean ± S.E.M. from at least six animals per group. Asterisks (*, *p* ≤ 0.05; **, *p* < 0.01) indicate significant differences between the treatment and controls unless otherwise indicated, while hashtags (##, *p* < 0.01) indicate significant differences between the indicated treatment groups.

After the motor deficit measurements, we assessed the effect of Mn, V_2_O_5_, and Mn/V_2_O_5_ on olfaction by measuring the social discrimination ability of male mice to detect female pheromones. Specifically, we evaluated their ability to find and sniff bedding from female cages, as described in the Methods section. Our results demonstrated a significant decrease in the time spent sniffing the female bedding during a 3-min testing session following the introduction of the bedding into the male mouse cages for the Mn, V_2_O_5_, and Mn/V_2_O_5_ treatment groups compared to the control group (Figure 2A). We observed a 36%, 66%, and 73% decrease in the time spent sniffing for the Mn, V_2_O_5_, and Mn/V_2_O_5_ treatment groups, respectively, relative to the controls. Furthermore, we observed a significant loss in the sense of smell in the Mn/V_2_O_5_ co-treatment group compared to the Mn-treated group. These findings indicate that prolonged intranasal exposure to these metals causes male mice to experience impaired olfactory function, with the degree of deficits in olfaction following this order: Mn/V_2_O_5_ > V_2_O_5_ > Mn.

### 2.3. Chronic Intranasal Exposure to Mn, V_2_O_5_, or Their Mixture (Mn/V_2_O_5_) Reduces the Weight of Olfactory Bulbs

To determine the effect of intranasally administered Mn, V_2_O_5_, and Mn/V_2_O_5_ on OB weight, we dissected and weighed the OBs, as described in the Methods section. Our results show a significant reduction in OB weight in the Mn, V_2_O_5_, and Mn/V_2_O_5_ combined treatment groups, with reductions of 21%, 34%, and 36%, respectively, compared to the control group (Figure 2B). Morphological changes in the OB (Figure 2C) confirmed the weight loss observed in Figure 2B. The total amount of protein (Figure 2D), as measured by the Bradford assay, also supports the observations in Figure 2B,C. These results indicate that chronic intranasal exposure to neurotoxic metals reduced OB weight and volume as well as olfactory function in male mice, with the severity in the following order: Mn/V_2_O_5_ ≥ V_2_O_5_ > Mn.

### 2.4. Chronic Intranasal Exposure to Mn, V_2_O_5,_ or Their Mixture (Mn/V_2_O_5_) Causes the Loss of Tyrosine Hydroxylase (TH) and Dopaminergic Neurons in the Olfactory Bulb

**Figure 3:**
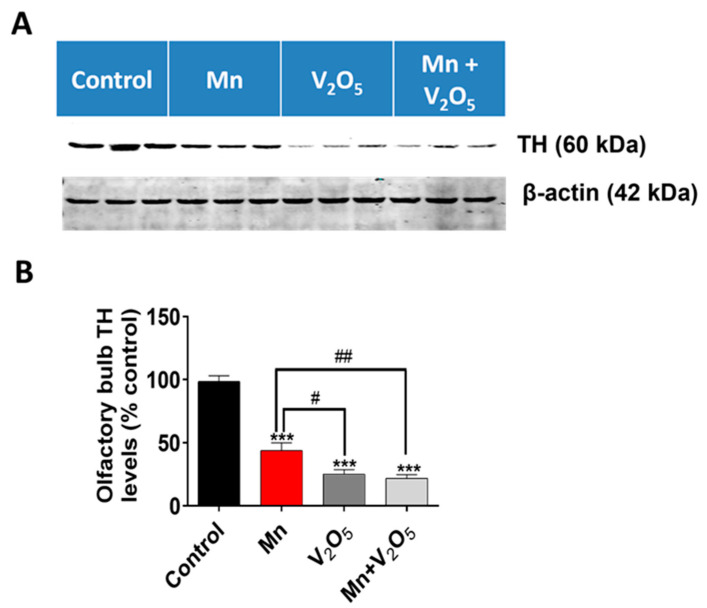
Effect of intranasally administered Mn, V_2_O_5_, and Mn/V_2_O_5_ on TH expression level in the OB. (**A**), Tissues of the OB were homogenized and used for Western blotting, as described in the Methods section. TH expression was detected using a monoclonal mouse antibody raised against TH. The membrane was re-probed with the β-actin antibody to confirm equal protein loading in each lane. (**B**), Densitometric analysis of the Western blot. The data represent the mean ± S.E.M. from at least seven animals per group. Asterisks (***, *p* < 0.001) indicate a significant difference between the treatment and control unless otherwise indicated, while hashtags (#, *p* ≤ 0.05; ##, *p* < 0.01) indicate significant differences between the indicated treatment groups.

**Figure 4:**
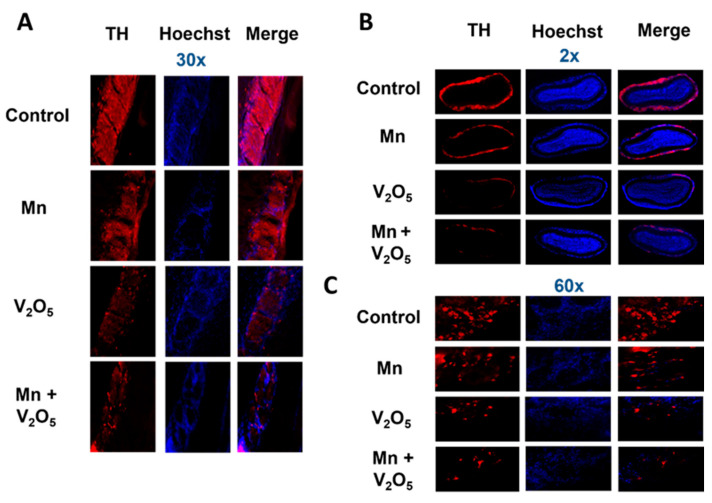
Loss of TH neurons in the OB following intranasally administered Mn, V_2_O_5_ alone, or Mn/V_2_O_5_ mixed. (**A**), Representative 30× pictures of TH neurons in the OB of the control and treated mice. (**B**), Representative 2× pictures of TH neurons in the OB of control and treated mice. (**C**), Representative 60× pictures of TH neurons in the OB of control and treated mice. Data were derived from 3 mice per treatment group.

It has been demonstrated that, as the initial synaptic integration site of the whole OB system, the glomerular layer of the OB is rich in dopaminergic neurons [48,49,50]. These neurons are linked with modulating olfactory signal processing since DA is thought to play an important role in proper olfactory function [51,52,53]. TH, which is the rate-limiting enzyme during DA synthesis, is often used as a marker for the presence of healthy dopaminergic neurons. We, therefore, measured the metal-induced changes in TH protein levels and immunoreactivity in the dissected OBs using Western blot (Figure 3) and immunohistochemistry (IHC) (Figure 4). TH protein levels in the OBs of metal-treated groups were significantly reduced relative to the control group (Figure 3A). The densitometric analysis of Western blot revealed a 56%, 74%, and 78% decrease in TH levels in the OBs of the Mn, V_2_O_5_, and Mn/V_2_O_5_ treatment groups, respectively, compared to the controls (Figure 3B). Furthermore, both V_2_O_5_-alone and Mn/V_2_O_5_ co-treatment groups showed significantly higher decreases in TH levels than Mn-alone. To provide further anatomical visualization of dopaminergic neurons in the glomerular layer of the OB, we performed a TH IHC analysis. Both V_2_O_5_ and Mn/V_2_O_5_ groups showed a greater loss of TH-positive neurons (Figure 4A–C), further supporting the Western blot data presented in Figure 3. Taken together, our findings suggest that chronic intranasal exposure to these metals, either alone or mixed, can cause significant neurotoxic effects in dopaminergic OB neurons.

### 2.5. Chronic Intranasal Exposure to Mn, V_2_O_5_, or Their Mixture (Mn/V_2_O_5_) Causes the Loss of the Neurotransmitter Dopamine and Its Metabolite 3,4-Dihydroxyphenylacetic Acid (DOPAC) in the Olfactory Bulb

**Figure 5:**
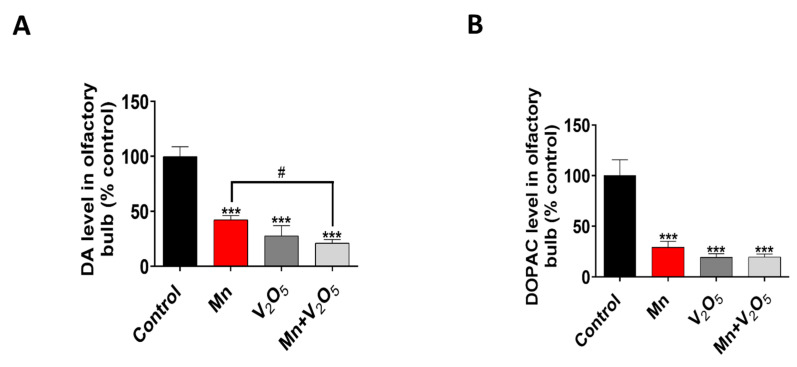
Effects of intranasally administered Mn, V_2_O_5_, and Mn/V_2_O_5_ on neurochemical changes in the OB. (**A**), DA levels and (**B**), DOPAC levels using HPLC. The data represent the mean ± S.E.M. from at least six animals per group. Asterisks (***, *p* < 0.001) indicate a significant difference between the treatment and controls unless otherwise indicated, while the hashtag (#, *p* < 0.05) indicates a significant difference between the indicated treatment groups.

Following the assessment of neurobehavioral and neuropathological changes associated with metal-induced impairment in the olfactory system, we further measured the neurochemical changes in the OBs to determine behavioral and neurochemical correlations. Our HPLC-ECD neurochemical analysis revealed a significant reduction in DA and DOPAC levels in the OBs of the Mn, V_2_O_5_, and Mn/V_2_O_5_ treatment groups, with a corresponding 57%, 72%, and 79% reduction in DA levels, and a 70%, 80%, and 80% decrease in DOPAC levels, relative to the control group (Figure 5A,B). The Mn/V_2_O_5_ treatment group showed significantly lower DA levels than the Mn group (Figure 5A,B). Collectively, these results show that exposure to Mn and V_2_O_5_, both alone and mixed, induces a profound dopaminergic neurochemical loss in the OB.

### 2.6. Exposure to Mn and V_2_O_5_ Mixture Results in a Higher Uptake of Both Metals in the Striatum

**Figure 6:**
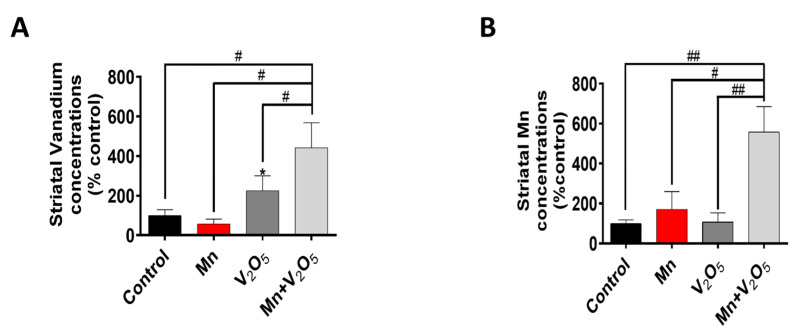
Striatal levels of Mn and V following intranasal administration of Mn, V_2_O_5_, or Mn/V_2_O_5_. (**A**), Mn levels and (**B**), V levels using ICP-MS. The levels were normalized to striatal tissue weight. The data represent the mean ± S.E.M. from at least four animals per group. The asterisk (*, *p* ≤ 0.05) indicates a significant difference between the treatment and controls, while hashtags (#, *p* ≤ 0.05; ##, *p* < 0.01) indicate significant differences between the indicated treatment groups.

Next, to ensure that intranasal administration resulted in the uptake of metals by the brain, we measured Mn and V metal levels in the STR using inductively coupled plasma mass spectrometry (ICP-MS). We observed a 4-fold increase in V levels in the STR of the Mn/V_2_O_5_ group compared to a 2-fold increase in the V_2_O_5_-alone group, both relative to controls (Figure 6A). We also observed a 5-fold increase in Mn levels in the STR of the Mn/V_2_O_5_ group compared to a 1.7-fold increase in the Mn-alone group, both relative to the controls (Figure 6B). These findings suggest that an Mn/V_2_O_5_ mixture leads to the greater uptake of both metals in the STR compared to their individual exposures.

### 2.7. Chronic Intranasal Exposure to Mn, V_2_O_5_, or Their Mixture (Mn/V_2_O_5_) Induces Oxidative Stress in the Striatum and Sustantia Nigra

**Figure 7:**
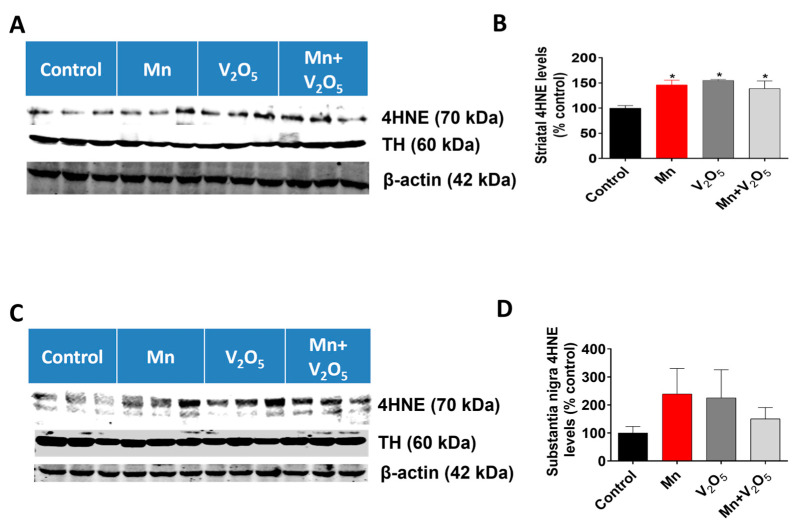
Effects of intranasally administered Mn, V_2_O_5_, and Mn/V_2_O_5_ on 4HNE expression levels in the STR and SN. (**A**), Striatal tissue was homogenized and used for Western blotting, as described in the Methods section. 4HNE and TH were detected using antibodies raised against 4HNE and TH, respectively. The membranes were re-probed with the β-actin antibody to confirm equal protein loading in each lane. (**B**), 4HNE densitometric analysis of the Western blot. (**C**), SN tissue was homogenized and used for Western blotting, as described in the Methods section. 4HNE and TH expressions were detected using an antibody raised against 4HNE and TH, respectively. The membranes were re-probed with the β-actin antibody to confirm equal protein loading in each lane. (**D**), 4HNE densitometric analysis of the Western blot. The data represent the mean ± S.E.M. from at least five animals per group. Asterisks (*, *p* ≤ 0.05) indicate significant differences between the treatment and controls unless otherwise indicated.

Since oxidative stress has been shown to be an early event during Mn-induced neurotoxicity, we measured the accumulation of the oxidative stress marker 4-hydroxynonenal (4HNE) in the STR and SN using Western blot analysis. We observed a 1.5-, 1.5-, and 1.4-fold increase in 4HNE levels in the STR of animals treated with either Mn, V_2_O_5_, or Mn/V_2_O_5_, respectively, compared to the controls (Figure 7A,B). Similarly, we observed a 2.4-, 2.3-, and 1.5-fold increase, although not statistically significant, in 4HNE levels in the SN of animals treated with either Mn, V_2_O_5_, or Mn/V_2_O_5_, respectively, compared to the control group (Figure 7C,D). TH levels in both the SN and the STR did not change, indicating that dopaminergic neurons or their processes in the SN and STR had not yet suffered severe damage.

### 2.8. Chronic Intranasal Exposure to Mn, V_2_O_5_, or Their Mixture (Mn/V_2_O_5_) Induces the Accumulation of α-Synuclein in the Substantia Nigra

**Figure 8:**
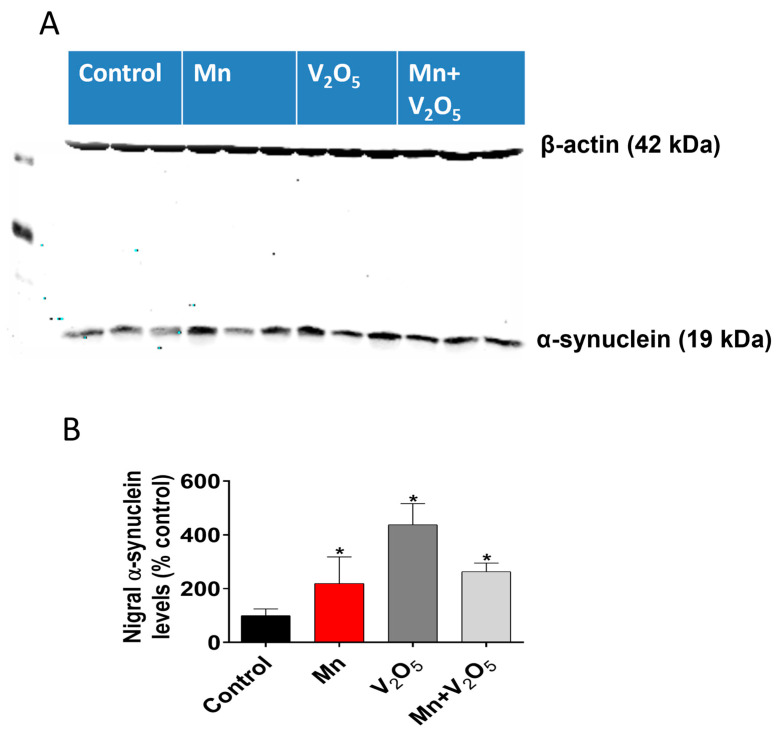
Effects of intranasally administered Mn, V_2_O_5_, and Mn/V_2_O_5_ on α-synuclein expression levels in the SN. (**A**), SN tissue was homogenized and used for Western blotting, as described in the Methods section. Alpha-synuclein expression was detected using a monoclonal antibody raised against α-synuclein. The membrane was re-probed with the β-actin antibody to confirm equal protein loading in each lane. (**B**), Densitometric analysis of the Western blot. The data represent the mean ± S.E.M. from at least five animals per group. Asterisks (*, *p* ≤ 0.05) indicate significant differences between the treatment and controls unless otherwise indicated.

The accumulation of α-synuclein in the SN has been shown to be an early event during dopaminergic neurodegeneration. Hence, we proceeded to measure the accumulation of α-synuclein using Western blots. Remarkably, our results showed a significant increase in α-synuclein accumulation in the SN of animals treated with Mn, V_2_O_5_, or Mn/V_2_O_5_, with a 2.2-, 4.2-, and 2.6-fold increase, respectively, compared to the control group, as measured by the densitometric analysis of Western blots (Figure 8A,B). These findings suggest that chronic intranasal exposure to these neurotoxic metals, either alone or mixed, triggers α-synuclein accumulation in the SN, which could contribute to the pathogenesis of metal-induced chronic Parkinsonism.

### 2.9. Chronic Intranasal Exposure to Mn, V_2_O_5_, or Their Mixture (Mn/V_2_O_5_) Induced Upregulation of the Pro-Apoptotic Kinase PKCδ in the Olfactory Bulb

**Figure 9:**
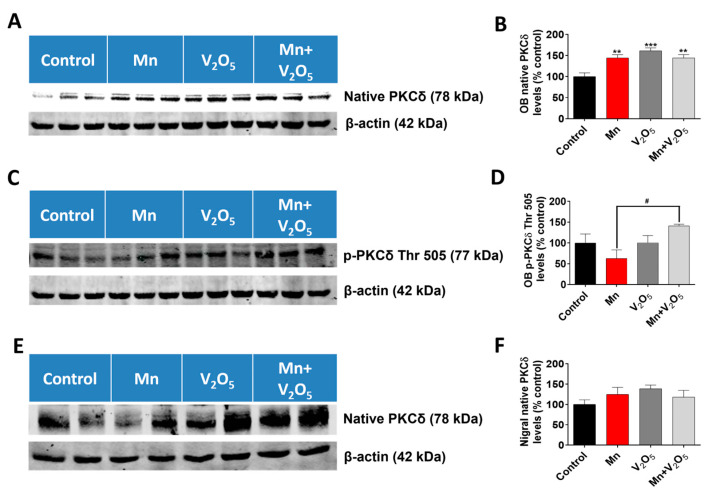
Effects of intranasally administered Mn, V_2_O_5_, and Mn/V_2_O_5_ on native PKCδ and PKCδ Thr 505 expression levels in the OB and SN. (**A**), OB tissue was homogenized and used for Western blotting, as described in the Methods section. PKCδ expression was detected using a monoclonal antibody raised against PKCδ. The membrane was re-probed with the β-actin antibody to confirm equal protein loading in each lane. (**B**), Densitometric analysis of the Western blot. (**C**), OB tissue was homogenized and used for Western blotting, as described in the Methods section. PKCδ Thr 505 expression was detected using a monoclonal antibody raised against PKCδ Thr 505. The membrane was re-probed with the β-actin antibody to confirm equal protein loading in each lane. (**D**), Densitometric analysis of the Western blot. (**E**), SN tissue was homogenized and used for Western blotting, as described in the text. PKCδ expression was detected using a monoclonal antibody raised against PKCδ. The membrane was re-probed with the β-actin antibody to confirm equal protein loading in each lane. (**F**), Densitometric analysis of the Western blot. The data represent mean ± S.E.M. from at least five animals per group. Asterisks (**, *p* < 0.01; ***, *p* < 0.001) indicate significant differences between the treatment and control unless otherwise indicated, while the hashtag (#, *p* ≤ 0.05) indicates a significant difference between the indicated treatment groups.

We demonstrated in our laboratory that the pro-apoptotic kinase PKCδ, a member of the novel subfamily of PKC kinases, is critical during metal-induced dopaminergic neurodegeneration [37,47,54]. We, therefore, measured the levels of this protein in the OB with SN as an indicator of ongoing apoptotic events using Western blots. We observed a significant 1.4-, 1.6-, and 1.4-fold increase in native PKCδ levels in the OB lysates following treatment with Mn or V_2_O_5_ alone, or with Mn/V_2_O_5_, respectively, compared to the controls, as measured by the densitometric analysis of Western blots (Figure 9A,B). Additionally, Western blot analysis showed a 1.4-fold increase in the levels of active PKCδ with phosphorylation at the threonine 505 site in the OB lysate following Mn/V_2_O_5_ co-exposure compared to the control group, while the treatment groups with Mn and V_2_O_5_ alone showed no significant differences (Figure 9C,D). In the SN, we also observed a modest, statistically insignificant increase in native PKCδ levels following treatment with Mn or V_2_O_5_ alone, or with Mn/V_2_O_5_, respectively, compared to the control group (Figure 9E,F). These results suggest that the upregulation of pro-apoptotic PKCδ might be involved in mediating the neurotoxic effects induced by these neurotoxic metals in the OB.

### 2.10. Chronic Intranasal Exposure to Mn, V_2_O_5_, or Their Mixture (Mn/V_2_O_5_) Causes Astrocytes to Accumulate in the Glomerular Layer of the Olfactory Bulb

**Figure 10:**
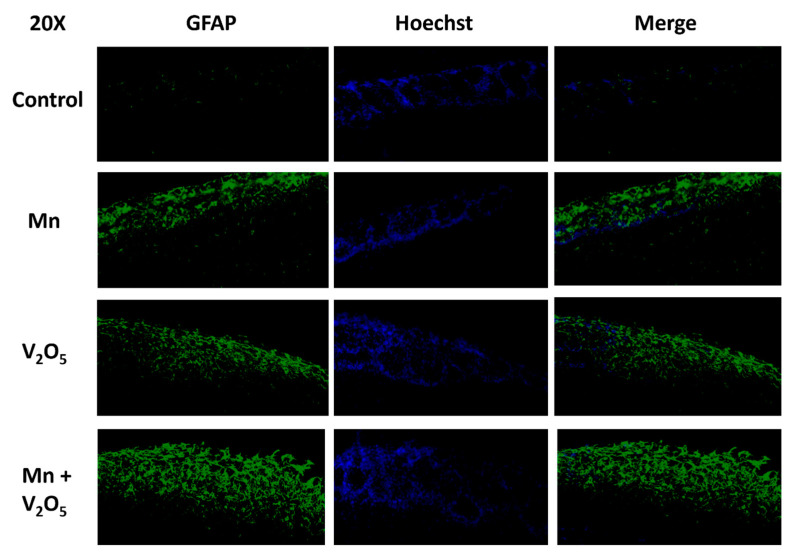
Astrocyte accumulation in the glomerular layer of the OB following the intranasal administration of Mn or V_2_O_5_ alone or Mn/V_2_O_5_ mixed. The astrocytes in the OB were identified by staining with GFAP using IHC and were found to accumulate in the glomerular layer following metal exposure. This figure shows representative 20× pictures of GFAP neurons in the OB of control and treated mice. Data were derived from 3 mice per treatment group.

We have shown that dopaminergic neurons in the glomerular layer of the OB degenerate following chronic exposure to neurotoxic metals (Figure 4A–C). Therefore, we examined astrocyte activation as a marker of the neuroinflammatory response in the glomerular layer of the OB using glial fibrillary acidic protein (GFAP) immunostaining. Our results show no GFAP immunoreactivity in the control group; however, a dramatic accumulation of astrocytes occurred in the glomerular layer of the Mn, V_2_O_5_, and Mn/V_2_O_5_ exposure groups (Figure 10). These findings suggest that metal-induced olfactory damage is associated with astroglial hyperactivation.

## 3. Discussion

In this study, we provide evidence for the first time that chronic exposure to low doses of Mn alone, V alone, or a Mn/V mixture via the intranasal route can induce olfactory dysfunction and adversely affect the nigrostriatal system. Observed metal exposure-induced olfactory neurotoxicity was accompanied by reduced OB weights, dramatically decreased DA and DOPAC levels, decreased levels of TH, the accumulation of astroglia in the glomerular layer, and loss of dopaminergic neurotransmission to the OB. Additionally, we observed that chronic exposure to Mn, V, or their mixture via the intranasal route induced locomotor deficits. Our results tend to show a greater severity in the Mn/V mixture group, with Mn alone inducing less severe adverse effects than V alone. Oxidative stress, as measured by the increased levels of 4HNE, increased in the STR. Interestingly, the increased expression of α-synuclein also occurred in the SN following the treatments, suggesting that chronic intranasal exposure to neurotoxic metals can upregulate the key pathological protein involved in PD. Increased Mn and V levels accumulated in the STR during Mn/V_2_O_5_ co-exposure compared to exposure to either metal alone. These exposures led to the upregulation of the pro-apoptotic kinase PKCδ in the OB. Our results show evidence of dopaminergic neuronal loss in the OB and not in the nigrostriatal system, suggesting that the olfactory system is adversely affected prior to the effects on the nigrostriatal systems, as has been suggested by Parkinsonism pathology in humans. Environmental factors are believed to contribute to the multifactorial etiology of Parkinsonism pathogenesis [55,56,57,58]. Occupational exposure to environmental neurotoxicants often occurs through inhalation and exposure to metals, which typically occurs in metal mixtures, which is a significant environmental risk factor for various neurodegenerative diseases, including PD [11,59,60,61,62]. Mn and V are both components of welding fumes generated during the welding process [43] and are present in various industries related to steel production, such as construction, car and aircraft manufacturing, and the production of temperature-resistant alloys and batteries, among others [43,63,64,65,66,67]. As the demand for high-capacity batteries grows in the electric vehicle industry, the use of V in developing such batteries grows due to the unique redox transfer property of V oxides [68,69,70]. Recent studies show that highly favorable V chemistry can be adapted to develop high-power and long-life energy storage devices [71].

Chronic exposure studies have ascertained the possible role of Mn in the etiopathogenesis of PD [72,73,74,75,76]. Similarly, Avila-Costa’s group and our lab have shown that V can damage the nigrostriatal dopaminergic systems and different components of the CNS in rodents [37,39,40,41], suggesting that V might also play a role in PD pathology and etiology. The concentrations we used in the current study are comparable to those used in previous studies [39,41,77]. Although strong evidence links welding fumes containing heavy metals, including Mn and V, to an increased risk for the development of neurological diseases, including PD [59,60,78,79,80], epidemiological studies linking metal mixtures to PD have been less certain in their conclusions. Some of these studies, for example, have reported a synergistic effect between Mn and Fe co-exposure and PD [81], even though no studies have shown an association with PD following exposures to Fe alone [82]. Correlations between metal mixture exposure and PD have also been reported for different combinations of Fe and Pb [82,83,84] and Fe and Cu [82,83,84]. Although increased levels of zinc (Zn) have been reported in the brains of PD patients [85,86], Gorell et al. [85] did not show any association between PD and Zn exposure. Co-exposure to oral doses of Mn and Pb can lead to synergistic neurological effects, as shown in rats by Chandra et al. [87], who reported significantly increased motor activity and neurotransmitter levels relative to rats exposed to only one metal. Mn/Pb co-exposed rats showed a greater deficit in learning conditioned avoidance responses compared to those exposed to either metal alone. Additionally, gestational co-exposure to Mn/Pb reduced brain weight more than either metal alone [88]. Exposure to mixtures of Pb, Mn, and arsenic (As) led to even greater changes in neurotransmitter levels in rat brains [89]. It has also been demonstrated that co-exposure to Mn and Pb increases the levels of Pb in the brain approximately three-fold compared to Pb-alone exposure, possibly due to co-exposure-related changes in the affinity of Pb-binding proteins in the brain [90,91]. In the current study, we observed increased striatal levels of Mn and V concentrations during Mn/V co-exposures relative to animals with exposures to either metal alone or in the control group. This finding supports previous research indicating that mid-brain structures critical for motor control, such as the STR and globus pallidus, are targets of Mn neurotoxicity and accumulated Mn [19]. In further support of our observation, Mn and As have also been shown to accumulate more in rat brains with Mn/As co-exposure treatment when compared to single metal exposure controls [92].

Oxidative stress has been shown to be a hallmark of apoptosis in metal neurotoxicity, particularly in relation to Parkinsonism [54,93,94,95]. Even though oxidative stress is known to be a critical factor in PD pathogenesis, with increased oxidative burden expressed in the form of increased levels of the lipid peroxidation product 4HNE in the brains of PD patients when compared to controls [96], the exact role of oxidative stress in metal-induced neurotoxicity is unknown [97]. Our study demonstrates increased levels of 4HNE in both the STR and SN of treated animals relative to controls through Western blots. Our research group and others have also shown that PKCδ is a pro-apoptotic kinase in dopaminergic neurons and a proinflammatory kinase in microglia [98,99,100,101,102]. PKCδ is oxidative stress-dependent in the CNS [98,103,104]. Our laboratory demonstrated in vitro that both V [37] and Mn [47] are neurotoxic to dopaminergic neurons via a caspase-mediated pro-apoptotic PKCδ mechanism. In this study, we observed the upregulation of PKCδ in the OB following exposure to Mn and V alone and during their co-exposure, suggesting ongoing proapoptotic and pro-inflammatory processes in OB.

Olfactory function has been shown to be compromised by metal-induced neurotoxicity in the OB. Various parameters, such as odor recognition memory experiments, odor identification, threshold detection, and odor discrimination, have been used to investigate odor deficits in metal-induced toxicity [105]. Interestingly, PD patients have been reported to exhibit deficits in odor differentiation, detection, and identification [106,107,108]. OB volumes and weight tend to decline with an associated decrease in the sense of smell [109,110,111,112,113]. It has been suggested that the early onset of olfactory dysfunction in PD patients might primarily be due to a damaged OB [112]. Pathological changes have been reported in both the OB [114] and brain regions associated with proper olfaction [115,116,117,118] during postmortem studies in PD patients. A study carried out by Silveira-Moriyama et al. [118] supports the potential of olfactory deficits as a biomarker for progressive cortical atrophy in the first stages of PD. Their study also suggests that symptoms of olfactory dysfunction and a compromised nigrostriatal dopaminergic pathway present themselves concomitantly during the pathological process of PD, as seen in our study. Furthermore, olfaction dysfunction is associated with increased risks of developing motor deficits associated with PD [119]. Therefore, understanding the changes in the OB and recognizing its related behavioral impairments are important.

Our results show that the dopaminergic neurotransmitter system in the OB is adversely affected by exposure to either Mn, V, or a Mn/V mixture. These findings are consistent with those of other studies, which have shown the depletion of DA in the OB after exposure to the classic catecholaminergic neurotoxicants methamphetamine and amphetamine [120,121]. Additionally, another study demonstrated that intranasal irrigation in mice with either ZnSO_4_ and surgical deafferentation or axotomy in rats decreased levels of DA, DOPAC, TH enzyme activity, and OB weights [122]. They also showed clear correlations between reductions in TH, DA and DOPAC levels and OB weights [122]. In this study, we also observed that a reduction in OB TH levels correlated with the loss of OB DA and DOPAC levels as well as OB weights for the treatment groups, with the greatest severity in the Mn/V co-exposure treatment group. These findings provide further evidence for the detrimental effects of Mn and V exposure on the dopaminergic neurotransmitter system in the OB and suggest that the OB may be particularly vulnerable to these metals, highlighting the importance of further investigating the mechanisms underlying the observed effects.

The OB is made up of five layers, which comprise the subependymal, combined mitral and granule cells, external plexiform, and glomerular layers [123,124]. The glomerular layer has an abundant expression of dopaminergic neurons [48,49]. The results from our study indicate that intranasal exposure to either Mn, V, or their mixture can decrease both TH and DA levels in the OB. DA is believed to play an important role in olfaction [51,125,126,127,128], and changes in DA levels affect olfaction. It has been observed that DA mediates the entry of olfactory information into the brain by inhibiting the transmission between the olfactory epithelium of the OB and the OB glomeruli [51]. In this regard, our results indicate that DA depletion is accompanied by a significantly decreased amount of time spent sniffing female bedding as a measure of hyposmia in all the treatment groups relative to the control. OB DA depletion following intranasal exposure to Mn, V, or their mixture may, therefore, impair pheromonal olfaction since, as previously stated, the disinhibition of olfactory glomeruli can be caused by low DA levels, leading to improperly organized olfactory processing. This supports clinical observations that PD patients experience hyposmia, typically preceding motor deficits [129].

Increased locomotor activity was reported in rats exposed to the Mn phosphate/Mn sulfate mixture [130] and Mn sulfate [131], though similar effects were not observed following exposure to Mn phosphate alone [132,133]. Another study reported decreased locomotor activity and impaired performance in a maze test of spatial memory in rats exposed to gavage doses of 6.5 or 25.9 mg Mn/kg/day for 10 weeks when compared with the controls [134]. Conversely, no changes in locomotor activity were observed in rats exposed to 11 or 22 mg Mn/kg/day for 21 days [135,136]. V exposure is known to cause severe motor deficits in humans [137,138]. Similarly, in this study, we did not observe any locomotor changes in the Mn treatment groups relative to the control. However, we observed significant locomotor deficits in the V-alone and Mn/V co-treatment groups. The olfactory and locomotor deficits tended to be most serious in the co-exposure group, while V alone induced more severe deficits than Mn alone.

The accumulation of abnormal and misfolded α-synuclein within neuronal cell bodies, axons, and synapses in the brains of sporadic PD patients and animal models of Parkinsonism is a pathological hallmark of the diseased state [139,140,141,142,143]. The potential role of α-synuclein in disease progression and development has been strongly supported by genetic evidence that α-synuclein mutations or multiplication of the α-synuclein gene can lead to familial PD [144,145]. Pathological changes attributed to α-synuclein usually occur during the early stages of the disease [115,146]. In this study, we observed an increase in protein levels of α-synuclein in the SN of the brain of metal-treated mice.

While we observed the highest effects in most measurements from the co-exposure group compared to the individual metal exposure groups, our data did not reveal strict additive or synergistic effects for co-exposure to Mn and V. This observation may be attributed to several factors, including differences in the mechanisms of action and the toxicokinetics of Mn and V. These metals may interact differently at the cellular and molecular levels, resulting in diverse responses in various endpoints. Additionally, the non-linear nature of metal toxicology could contribute to the variability of our results. Our goal was to investigate the combined effects comprehensively, and the results presented here reflect the actual outcomes observed under our experimental conditions.

Overall, our results show that V is a more potent neurotoxicant than Mn in inducing damage to OB and nigral dopaminergic systems. Mn/V mixtures induce more pronounced neurotoxic responses compared to individual metal exposure. Notably, intranasal exposure to these metals not only affects the olfactory system but also induces pathological changes in the SN, such as α-synuclein upregulation. Metal exposure also increases oxidative stress in the nigrostriatal system. The growing co-usage of Mn and V in different applications only increases the risk of being environmentally co-exposed to both metals. It is, therefore, invaluable to probe their possible neurotoxic interactive properties. Since it has previously been shown in vitro, ex vivo, and in vivo that V [37,39,40,41] and Mn [47,73] are dopaminergic neurotoxicants, the findings in this study suggest a greater neurotoxic outcome following exposures to Mn/V mixtures when compared to either metal alone. This study expands our understanding of possible metal mixture toxicology relative to individual metals in the brain. Future studies should explore the mechanistic pathways underlying the observed neurotoxic effects, particularly examining the role of other potential modulatory neurotoxic metals and their synergistic interactions. Additionally, longitudinal studies could elucidate the long-term impacts of Mn and V exposure on olfactory function and its correlation with neurodegenerative diseases, enhancing our understanding of disease progression and prevention strategies.

## 4. Materials and Methods

### 4.1. Materials

We purchased manganese chloride (MnCl_2_, Cat. No. 203734), vanadium pentoxide (V_2_O_5_, Cat. No. 204854), protease cocktail, and phosphatase inhibitors (Cat. No. 1861281) from Sigma (St. Louis, MO, USA). The Bradford protein assay kit was obtained from Bio-Rad Laboratories (Hercules, CA, USA). Perchloric acid (Cat. No. 244252), EDTA (Cat. No. 79884), DA (Cat. No. PHR1090), and DOPAC (Cat. No. 11569) were purchased from Millipore Sigma (Burlington, MA, USA). NaS_2_O_5_ (Cat. No. S244) was purchased from Fisher (Hampton, NH, USA). The primary antibodies used in this study were as follows: anti-mouse tyrosine hydroxylase (TH) obtained from Millipore (Billerica, MA, USA, Cat. No. MAB318); the anti-mouse glial fibrillary acidic protein (GFAP) purchased from Cell Signaling Technology, Inc. (Danvers, MA, USA); anti-mouse 4HNE acquired from R&D Systems, Inc. (Minneapolis, MN, USA, Cat. No. MAB3249); anti-mouse α-synuclein (Cat. No. 610787) and anti-mouse native PKCδ (Cat. No. 610397) obtained from BD Biosciences (Palo Alto, CA, USA); anti-rabbit phospho-PKCδ purchased from Invitrogen (Waltham, MA, USA, Cat. No. PA5–17903); and anti-β-actin purchased from Sigma (Cat. No. A5441). The Alexa 680-conjugated anti-mouse and IRDye800-conjugated anti-rabbit secondary antibodies were purchased from Invitrogen and Rockland Inc., respectively.

### 4.2. Locomotor Activity

We performed open-field locomotor behavioral experiments both pre- and post-treatment using VersaMax animal activity monitors (model RXYZCM-16; AccuScan Instruments Inc., Columbus, OH, USA), as described in our previous studies [38,147,148,149,150]. The mice were placed in the middle of a transparent Plexiglas chamber for a 12-min test session covered with a ventilated Plexiglas lid. The first two minutes were for acclimatization to reduce the novelty effect, and the last 10 min were analyzed. The data are expressed as a percentage of the de-ionized water-treated control group.

### 4.3. Animals

A total of 48 (12 mice/group) male C57BL/6 mice aged six to eight weeks were housed in standard conditions using procedures approved and supervised by the Institutional Animal Care and Use Committee (IACUC protocols 12-5-6042, 18-309, 18-321) at Iowa State University (Ames, IA, USA). Based on the amounts used in a published study [39] and our pilot experiments, we intranasally administered MnCl_2_ alone (252 µg), V_2_O_5_ alone (197.91 µg), or a mixture of MnCl_2_ (252 µg) and V_2_O_5_ (197.91 µg) to mice three times a week for up to 4 weeks. The metals were administered intranasally in a 50 µL volume to mice using micropipettes after briefly anesthetizing the mice with isoflurane to prevent a gag reflex. Vehicle control groups received an equal volume of de-ionized water. Intranasal delivery was preferred because it took advantage of the olfactory epithelium’s incomplete blood-brain barrier (BBB) [151]. The olfactory nerves bypass the BBB, enabling chemicals to be taken up by these neurons and transported directly into the brain [151]. This route of administration is similar to the inhalation of airborne contaminants that occur during human occupational exposures. Following the treatments, mice were dissected (8 mice/group) after euthanasia via CO_2_ or transcardially perfused (4 mice/group) with a 4% solution of paraformaldehyde after being deeply anesthetized with a cocktail of ketamine (200 mg/kg) and xylazine (20 mg/kg) to carry out neurochemical and histological studies. Freshly dissected tissues were stored at −80 °C prior to analyses. Perfused brains were immediately post-fixed with PFA and 30% sucrose, then cryosectioned into 30-μm coronal sections using a cryostat and kept at −20 °C in a 30% sucrose–ethylene glycol solution.

### 4.4. Weighing Olfactory Bulbs

As previously described [122], we dissected the OB at the junction between the OB and the rest of the brain during the dissection of the mouse brains for various parts of interest. After dissection, the OB was weighed using an electronic balance according to the manufacturer’s guide and good laboratory practice prior to analysis.

### 4.5. Olfaction Test

The function of intact olfaction was measured by assessing the mouse’s ability to detect and sniff pheromones from female bedding. We combined the principles of the wooden block test, which leverages the ability of mice to discriminate between self- and non-self-odors [152,153] and the fact that the body odor and urine of female rodents contain pheromones that attract males [154,155]. Specifically, a small quantity (approximately the same for every animal) of bedding from a mouse cage housing pregnant females was introduced into the cages of the male mice under study during the week following the last day of treatments. The amount and location of bedding remained constant every time. During a three-minute testing session, the total amount of time that each mouse spent sniffing the foreign bedding was measured with the aid of a stopwatch. This test is an easy and effective way of assessing the olfactory capacity of the mouse to detect a foreign odor, in this instance, female pheromones.

### 4.6. HPLC Detection of Dopamine

To determine the levels of DA and its metabolite, DOPAC, in OB tissues, we employed a high-performance liquid chromatography method with electrochemical detection (HPLC-ECD). Using OB tissue dissected from the subgroup of mice identified in Section 4.3, the samples were prepared for analyses, as described in our previous publications [148,156]. Using an antioxidant extraction solution composed of 0.1 M of perchloric acid containing 0.05% Na_2_EDTA and 0.1% Na_2_S_2_O_5_, the neurotransmitters were extracted from the dissected OB on the day of analysis. The extracts were filtered in 0.22-μm spin tubes and diluted 1:2 in a commercial mobile phase (MDTM, Thermo-Scientific, Waltham, MA, USA). The DA and DOPAC from the extracts were separated isocratically using a reversed-phase HPLC C-18 column (ZORBAX Eclipse Plus 4.6 × 100 mm, Agilent, Santa Clara, CA, USA) with a flow rate of 0.7 mL/min. The HPLC-ECD system (ESA Inc., Bedford, MA, USA) was equipped with an automatic sampler with refrigerated temperature control (model 542; ESA Inc.). The ECD system comprises a Coulochem model 5100A with a microanalysis cell (model 5014A) and a guard cell (model 5020) (ESA Inc.). In total, 1 mg/mL standard stock solutions of catecholamines were prepared in the antioxidant extraction solution and diluted to a final working concentration of 50 pg/μL prior to injection. The data were acquired using EZStart HPLC Software version 7.2 (ESA Inc.) and analyzed in Excel 2013 (Microsoft, Redmond, WA, USA) and Prism 4.0 (GraphPad, San Diego, CA, USA), as described previously [147].

### 4.7. Western Blot

Lysates were prepared by homogenizing dissected SN, STR, and OB tissues from the control and treatment groups with a pestle using the modified RIPA buffer with phosphatase and protease inhibitors. The lysates from the different treatment groups were normalized to contain equal concentrations of the protein. Equal amounts of protein were loaded into each lane and separated on a 10 to 15% SDS-polyacrylamide electrophoresis gel, as described in previous studies [148,157,158]. Following gel electrophoresis, the proteins were transferred to a nitrocellulose membrane and blocked for 1 h with an Odyssey blocking buffer (LI-COR Biosciences, Lincoln, NE, USA) to prevent non-specific binding. The nitrocellulose membranes containing the transferred proteins were then incubated with the appropriate primary antibodies (1:1000), followed by incubation with either Alexa Fluor 680-conjugated anti-mouse IgG (1:10,000) or the IR800-conjugate anti-rabbit secondary antibody (1:10,000) for 1 h at room temperature (RT). The membranes were re-probed with a β-actin antibody (1:5000 dilution) to ensure the loading of equal protein in each lane. Western blot images were captured and analyzed using the Odyssey IR Imaging system (LI-COR Biosciences, Lincoln, NE, USA). We performed densitometric analysis on the appropriate bands of interest.

### 4.8. Immunohistological Analysis of Olfactory Bulb Sections

Immunolabeling was carried out on OB sections from the perfused brains described in Section 4.3. On the day of staining, stored sections were rinsed in PBS and blocked with 2% bovine serum albumin, 0.5% Triton X-100, and 0.05% Tween-20 in PBS for 1 h at RT. After blocking, the sections were incubated with the appropriate primary antibody overnight at RT. Following primary antibody incubation, the sections were washed with PBS several times (5× for 5 min each) and incubated with the appropriate secondary antibody for 90 min at RT. After this, the sections were washed with PBS again and incubated with 10 µg/mL Hoechst 33342 for 5 min at RT to stain the nucleus. The stained sections were carefully mounted on poly-L-lysine-coated slides with the DPX organic solvent, followed by dehydration through being kept for one min each in water, 70% ethanol, 95% ethanol, 100% ethanol, and Xylene in that order. The sections were imaged using an inverted fluorescence microscope (model TE-2000U; Nikon, Tokyo, Japan), and 2×, 20×, 30×, or 60× images were captured with a SPOT digital camera (Diagnostic Instruments, Inc., Sterling Heights, MI, USA) using MetaMorph, version 5.0 (Molecular Devices, Sunnyvale, CA, USA).

### 4.9. Measurement of Metal Levels

Mn and V levels in the STR were measured using ICP-MS in a similar method to that which we previously used to measure essential metal concentrations in N27 cells [37]. Striatal tissues dissected from treated mice were used for the analysis. We used ICP-MS to determine the metal concentrations at *m/z* 51 in each sample. To resolve the isotopes of interest from any interferences, we used the ELEMENT 1 ICP-MS device (Thermo Finnigan, San Jose, CA, USA), which is a high-resolution double-focusing instrument operated at medium resolution (m/∆m = 4000) [159]. The samples were each placed in an acid-washed 5-mL Teflon vial and digested in 150 L of high-purity nitric acid (Ultrex II, J.T. Baker). After digestion, the samples were diluted to 5 mL with 18.2 MΩ of deionized water to achieve a final acid concentration of approximately 3% nitric acid. The supernatant was then analyzed with the ICP-MS to obtain the metal concentrations. An internal standard method was used for quantification, as described previously [37,160]. The results for every sample were calculated using the integrated average background-subtracted peak intensities from 20 consecutive scans. To correct for differences in elemental ionization efficiency in the ICP-MS, a multi-element standard was used to derive normalization factors for V and Mn. Concentrations for V and Mn were subsequently calculated for each sample.

### 4.10. Data Analysis

Data analysis was performed using Prism 4.0 (GraphPad, San Diego, CA, USA). For all experiments, raw data were analyzed using one-way ANOVA. Statistically significant differences between the control and treatment groups or between treatment groups are indicated in figures by asterisks (* *p* ≤ 0.05, ** *p* < 0.01, and *** *p* < 0.001) or hashtags (# *p* ≤ 0.05, ## *p* < 0.01, and ### *p* < 0.001), respectively.

## Data Availability

The data presented in this study are available on request from the corresponding author.

## Abbreviations

Vanadium pentoxide, V_2_O_5_; vanadium, V; manganese, Mn; olfactory bulb, OB; substantia nigra, SN; cadmium, Cd; copper, Cu; Parkinson’s disease, PD; striatum, STR; dopamine, DA; central nervous system, CNS; tyrosine hydroxylase, TH; immunohistochemistry, IHC; 3,4-dihydroxyphenylacetic acid, DOPAC; 4-hydroxynonenal, 4HNE; glial fibrillary acidic protein, GFAP; iron, Fe; lead, Pb; zinc, Zn; arsenic, As; blood-brain barrier, BBB; room temperature, RT; inductively coupled plasma mass spectrometry, ICP-MS.
